# Bio-Inspired Aquatic Propulsion Mechanism Using Viscoelastic Fin Containing Fiber Composite Shear Thickening Fluid

**DOI:** 10.3390/biomimetics8050405

**Published:** 2023-09-01

**Authors:** Shunichi Kobayashi, Kousuke Sugiyama

**Affiliations:** 1 Institute for Fiber Engineering, Shinshu University, Ueda 386-8567, Japan; 2 Graduate School of Science and Technology, Shinshu University, Ueda 386-8567, Japan

**Keywords:** aquatic propulsion mechanism, elastic fin, shear thickening fluid

## Abstract

Many propulsion mechanisms utilizing elastic fins inspired by the caudal fins of aquatic animals have been developed. However, these elastic fins possess a characteristic whereby the rigidity required to achieve propulsion force and speed increases as the oscillation velocity increases. Therefore, by adding an actuator including a variable stiffness mechanism to the fin it is possible to maintain the optimal stiffness at all times. However, if the aforementioned characteristics allowing the fin itself to change stiffness are present, the need for a variable stiffness mechanism is eliminated, leading to possibilities such as the simplification of the mechanism, improvements in fault tolerance, and enhancements in fin efficiency. The authors developed a fiber composite viscoelastic fin by adding fibers to a shear thickening fluid (STF) and examined the speed dependency of the fin’s rigidity. In this work, we examined the structure and speed dependency of the fin’s rigidity, as well as the propulsion characteristics in still water and in uniform flow. As a result, the fiber-containing fin containing the STF oobleck (an aqueous suspension of potato starch) demonstrated higher propulsion in still water and higher self-propelled equivalent speed in uniform water flow than elastic fins.

## 1. Introduction

The mechanism of propulsion within water has traditionally been achieved predominantly through the utilization of screw propellers, which are most commonly associated with maritime vessels such as ships. However, there are concerns about the safety and environmental impact of screw propellers, including hazards to aquatic organisms, due to their high-speed rotation, problems with the entrapment of fishing nets and seaweed, and the pollution of water bodies due to violent agitation in shallow waters [1,2]. To address such significant flaws, the swimming of aquatic lifeforms has drawn attention, and a propulsion mechanism in water based on the vibratory motion of soft tail fins, modeled after their swimming mechanisms, has been studied [3,4,5]. Regarding the design of flexible fins, which aim to mimic the biomechanics of soft caudal fins, it has been found that the optimal stiffness required for achieving the highest propulsion efficiency is not a constant variable. Instead, stiffness has been observed to vary based on a range of factors, including environmental conditions and the type of movement required. However, attempting to change the stiffness of the fin during oscillatory motion presents difficulties, as directly replacing fins with different stiffness values is not practical. To address this issue, the authors and their colleagues developed two types of variable-stiffness fin. For the first fin, an elastic fin composed of a plate spring was prepared, the plate spring was clamped to a rigid body, and the clamping length was varied to change the “effective length”, which is the length of the unclamped portion. The configuration was designed such that when the effective length of the plate spring is short, the bending stiffness is high, and when the effective length is long, the bending stiffness is low [6,7,8]. For the second fin, an elastic plate fin was prepared, and the bending stiffness was increased in the longitudinal direction of the elastic plate by twisting it. The elastic plate demonstrated increased stiffness when the twist was greater, and decreased stiffness when the twist was lessened [9]. Crucially, the findings from these investigative studies revealed that, as the oscillation velocity of the fin-like object increases, the stiffness deemed optimal for both the propulsive force and speed also increases. However, it has been found that a variable stiffness mechanism depending on the torsion of the elastic plate can achieve general flexible deformation, but it has limitations, in that it cannot freely change the distribution of the bending stiffness along the longitudinal direction of the elastic plate. In addition, there are other technical hurdles that need to be overcome, such as the waterproofing of the driving motors, which must be housed within a casing specifically designed for the mechanism, to realize the concept of variable-stiffness fins.

If fins could intrinsically exhibit this variable stiffness characteristic, they would effectively render the variable stiffness mechanism and its associated actuators obsolete. This consequently simplifies the entire mechanical system substantially, and also raises expectations about the potential improvement in the fault tolerance and efficiency of the fin mechanism. Therefore, in response to this challenge, the authors present a prototype of a viscoelastic fin that encapsulates a fiber composite infused with a shear thickening fluid. Shear thickening fluids are those whose viscosity properties increase as the shear rate, exerted by external forces, increases, thereby demonstrating behaviors closer to a solid. In our research, we utilized a suspension of water and potato starch to serve this purpose. This type of fin exhibited elevated stiffness at high shear rates and reduced stiffness at lower shear rates. The inclusion of fibers in the composite had the effect of further augmenting this speed-dependent stiffness. Furthermore, by manipulating the alignment of the fibers, an anisotropic stiffness property can be achieved [10]. Within this research, we thoroughly examined the propulsion characteristics of an underwater bio-inspired propulsion mechanism utilizing this novel fin. In this paper, we first present a description of the fin’s structure, followed by an examination of the speed dependence of its stiffness. Subsequently, we investigate the propulsion force by altering the oscillating motion period in a still water environment. Furthermore, in a uniform water flow state, we evaluate the self-propelled equivalent speed, assuming a swimming state by modulating the oscillating motion period.

## 2. Materials and Methods

### 2.1. Fiber Composite Viscoelastic Fin Containing Shear Thickening Fluid

#### 2.1.1. Concept of Bending Speed Dependence of Fin Stiffness

**Figure 1:**
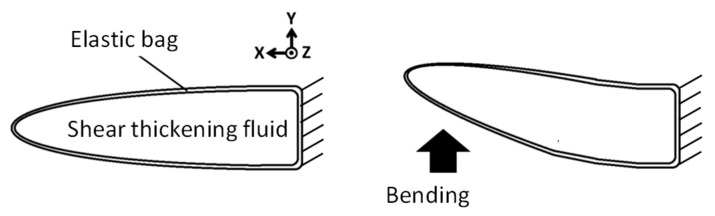
The concept of a viscoelastic fin containing a shear thickening fluid.

Shear thickening fluids are a type of non-Newtonian fluid where viscosity increases with an increase in the shear rate [11]. Figure 1 provides a visual representation of the correlation between fin rigidity and bending speed, thus offering valuable insights into the mechanical behavior. In instances where a bending force is exerted on the fin, the shear thickening fluid housed within the elastic bag begins to flow, which in turn precipitates an increase in the shear rate of the fluid. As the bending speed applied to the fin is increased, both the shear rate and the viscosity of the shear thickening fluid increase, subsequently leading to an escalated resistance against the bending force applied to the fin. This phenomenon means that the apparent bending stiffness of the fin appears high. Conversely, in scenarios where the bending speed applied to the fin is low, the flow velocity of the shear thickening fluid within the resilient elastic bag is correspondingly low. As a result, there is a consequent reduction in the viscosity of the shear thickening fluid, and therefore the resistance to the bending of the fin remains relatively low. This means that the apparent bending stiffness of the fin under these conditions appears low.

**Figure 2:**
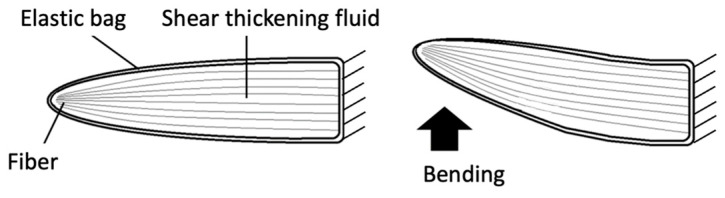
The concept of a viscoelastic fin containing a fiber composite shear thickening fluid.

Improvements were made to this system by introducing fibers into the shear thickening fluid within the fin. As shown in Figure 2, bundles of fibers are located inside the fin, with the right end of the bundle affixed to the fin, while the left end is not. As a result, when the fin is bent, sliding occurs between the fibers. Due to the minuscule distances that exist between each fiber, there is a larger gradient in the shear rate, which thereby promotes a significant increase in both the shear rate and the viscosity of the shear thickening fluid. Typically, shear thickening fluids are categorized as suspensions, and the added fibers function to prevent particle sedimentation, while simultaneously preserving the characteristic properties of the shear thickening fluid. Consequently, this fin shows the structure of a new fiber composite material, one that is uniquely equipped with the capacity to alter its rigidity [10].

#### 2.1.2. Structure of a Viscoelastic Fin Containing Fiber Composite Shear Thickening Fluid

**Figure 3:**
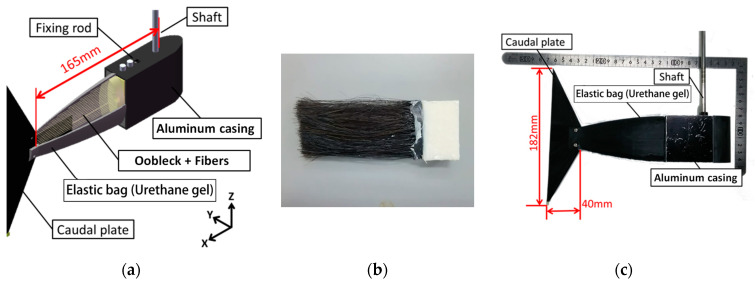
Viscoelastic fin containing a fiber composite shear thickening fluid. (**a**) Three-dimensional structure (**b**) Fibers inside the elastic bag. (**c**) External appearance.

Figure 3 provides a detailed visual representation of the design and outward form, or external morphology, of the viscoelastic fin. This fin was crafted using a composite of fibers and a substance known as a shear thickening fluid, which possesses viscoelastic properties. The design of the fin further comprises an aluminum casing, an elastic bag, and a caudal fin. Encased within the elastic bag, a mixture of the shear thickening fluid and fibers is carefully arranged. Moreover, a specifically constructed shaft is firmly attached to the aluminum casing, an addition that facilitates the fin’s rotational movement. The elastic bag consists of a soft urethane gel, which possesses a hardness measure of 40 on the Asker C scale (Young’s modulus equivalent to approx. 1 MPa), a scale commonly used for measuring the hardness of rubbery materials. In the process of developing the shape of the elastic bag, 3D-CAD was used. Utilizing the design data obtained, a 3D printer was utilized to fabricate the mold and core. Subsequently, a solvent and a curing agent specifically for soft polyurethane were cast into the resultant mold and core. This led to gelation, or the formation of a viscoelastic structure. In a frequency sweep test of a much softer Asker C scale 15 urethane gel performed by Nishino et al. using a rotational rheometer, the storage modulus increased from 160 to 170 kPa and the loss modulus increased from 16 to 22 kPa when the frequency was increased from 0.5 Hz to 2.0 Hz [12]. Thus, while the loss modulus has some effect, the storage modulus has a greater effect. It is also thought that the viscosity of oobleck, which is encapsulated in large quantities, would have a greater effect on the viscosity of a fin than the viscosity of soft urethane gel.

**Figure 4:**
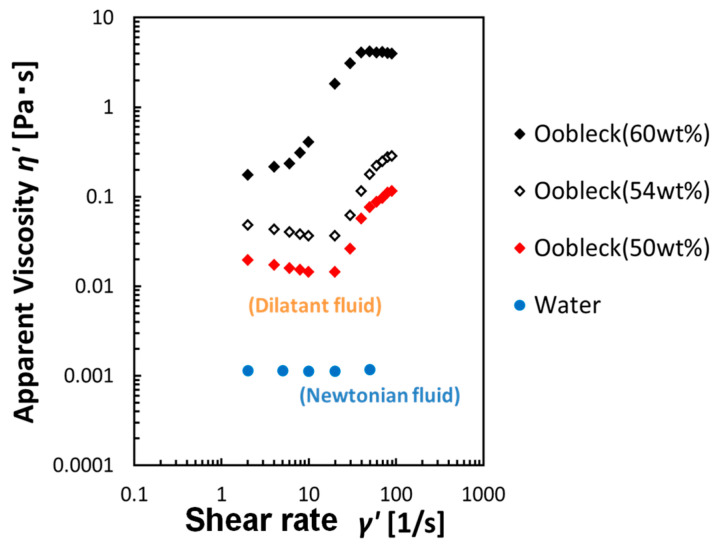
Relationship between the apparent viscosity and shear rate of fluid.

For the shear thickening fluid, oobleck, an aqueous suspension of potato starch, was selected and used [13,14,15]. Figure 4 illustrates the correlation between the fluid’s apparent viscosity and the shear rate, as measured with a rotational rheometer Rheologia A300 (Elquest, Chiba, Japan), a device designed to measure the flow properties of fluids. The data show that the apparent viscosity of the oobleck displayed a significant and sudden increase beyond a specific shear rate. To fabricate the fin, oobleck that contained 60 wt% potato starch was employed. For the fin’s fiber composition, a type of animal fiber typically used in paint-brushes and crafts, namely horsehair, was selected and incorporated. Table 1 provides a detailed enumeration of the fibers’ properties. Both hard and soft variants of these fibers were prepared and utilized.

Table 2 outlines various types of fin design. Each incorporates different substances within the confines of an elastic bag. For the fiber composite fluids, we constructed a blend of 60 wt% oobleck as a shear thickening fluid and fibers. Additionally, we also used a mixture of water as a Newtonian fluid and fibers, for the sake of comparison. To discuss the influence of fibers on performance, we prepared samples of 60 wt% oobleck and water without any fiber composites. We also made fins that were sealed with a uretFhane gel, due to its high elasticity, serving as the primary material. Other fin variations were made with a rigid acrylonitrile butadiene styrene (ABS) resin core, due to its rigidity, acting as the main structural component.

### 2.2. Experimental Methods to Examine the Bending Resistance of Fins

**Figure 5:**
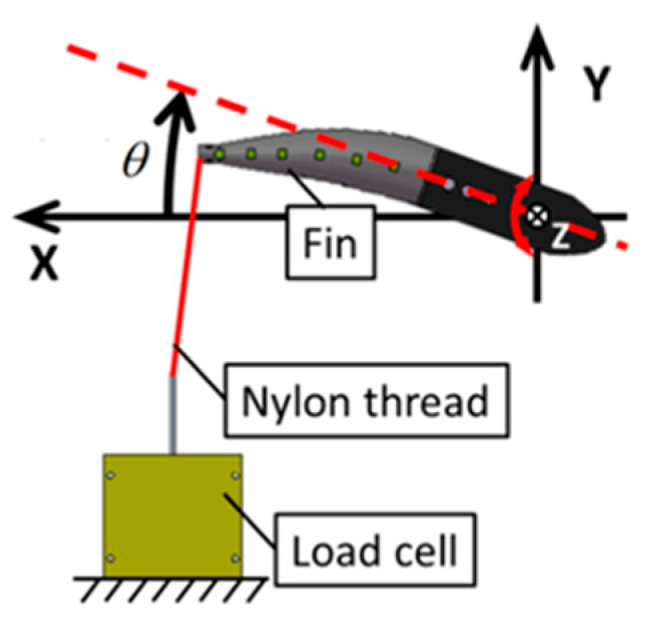
Schematic of the bending test. The red dashed line is the centerline of the aluminum casing (Figure 3).

Figure 5 shows a schematic design of a methodical bending test to assess the dependence of the fin’s bending velocity on its stiffness. The procedural setup to facilitate this evaluation involved a fin that was rotationally driven by a geared DC servo motor. This motor, a 118,755 (Maxon motor, Sachseln, Switzerland), was under the control of a LabVIEW system. The superior end of the fin was linked to a load cell T1-1000-240 (AND Orientec, Tokyo, Japan) via a nylon thread. This particular setup allowed detailed observation of the fin’s behavior under rotational force. When the fin experienced rotation, it responded by bending, and in the process, the nylon thread experienced a tension force. This force was measured using the aforementioned load cell, providing data about the fin’s dynamic behavior. The force that was measured was subsequently recorded as the bending resistance of the fin. Importantly, this bending resistance corresponds to the fin’s apparent dynamic stiffness. The fin’s oscillation angle, with respect to the time, underwent a sinusoidal variation. To initiate the process, the fin was initially displaced from an oscillation angle of negative 20 degrees. A tension force became apparent when the oscillation angle reached the zero-degree mark. Furthermore, the angular velocity of the fin’s oscillation peaked when the oscillation angle was exactly at 0°, and dropped to zero at the point of the maximum oscillation angle θ_max_. This maximum oscillation angle, θmax, for the fin was consistently maintained at 20° during the experiment. The average angular velocity was then derived through manipulating the oscillation period T of the fin. As part of the comprehensive study, we were also able to witness the fin’s bending behavior, thanks to the high-resolution footage captured using an HAS-220 high-speed video camera (capable of 200 fps, 640 × 480 pixel resolution, Ditect, Tokyo, Japan). Over the course of this experimental procedure, the fins underwent enforced deformation, imposing a significant load on the driving motor. This resulted in a situation where, even though the θ_max_ was strictly fixed at 20°, the material composition of the fin generated variation in the motion period T. These differing periods T remained within an acceptable range of 1.5 to 3.0 s. Additionally, the computed average angular velocity of the fin, denoted as ω, was seen to lie within a range of 0.35 to 0.9 rad/s. The bending resistance of the fin of the rigid ABS resin core was not evaluated because it did not deform.

### 2.3. Experimental Methods to Examine Propulsion Characteristics

#### 2.3.1. Still Water

**Figure 6:**
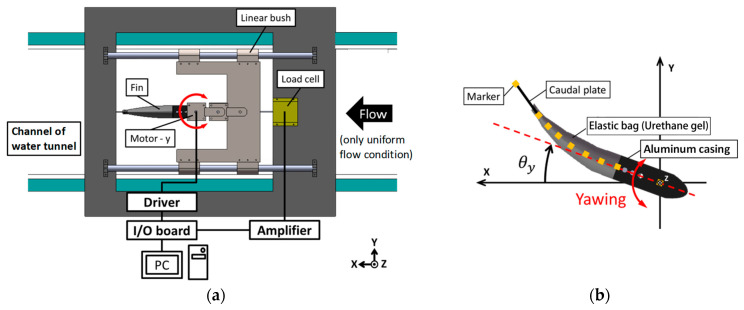
Measurement system for propulsion experiment. (**a**) Overall configuration; (**b**) Fin movement.

Figure 6a offers an illustrative diagram outlining the details of the experimental measurement setup for propulsion in water. The propulsion device was placed within the measurement channel of the water tunnel, specifically, a model PT-10 (West Japan Fluid Engineering Laboratory, Sasebo, Japan). The measurement pathway in the water tunnel had the dimensions of 30 cm in height, a width of 30 cm, and a length of 100 cm. The water depth within this system was maintained at 20 cm. In this measurement channel, the fin was positioned centrally, both along the horizontal and longitudinal planes. For the duration of the experiment, which was conducted in still water, the fin was not subjected to uniform fluid flow. The yawing motions of the fin were actuated by a geared DC servo motor 118,755 (Maxon motor, Sachseln, Switzerland), and controlled with the assistance of LabVIEW software. The propulsion device was securely attached to two linear guides, facilitating the device’s movement along the *x*-axis, and connected to a load cell, T1-1000-240 (AND Orientec, Tokyo, Japan), measuring the thrust force in the x direction. Visual tracking of the fin’s behavior, as well as video documentation of the experiment, were achieved using the HAS-220 high-speed video camera (capable of 200 fps, 640 × 480 pixel resolution, Ditect, Tokyo, Japan). This camera was placed below the measurement channel, to avoid any refraction of light on the water surface that could distort the video recordings. As an additional measure and as delineated in Figure 6b, we attached nine markers to the fin to provide a better understanding of its motion. The coordinates of these markers in the recorded videos were determined using the 2D video measurement software Move-tr/2D (Library, Tokyo, Japan). The experimental conditions are concisely summarized in Table 3.

#### 2.3.2. Uniform Water Flow

The experimental arrangement and the conditions were equivalent to the stationary water environment, as delineated in Figure 6 and Table 1. In the experiments, which were conducted under a uniform flow of water, data related to the self-propelled equivalent speed were procured. The term “self-propelled equivalent speed” refers to the velocity achieved by manipulating the water flow speed in a water tunnel to such an extent that the average propulsive force generated by the propulsion mechanism becomes zero. Consequently, this speed is equivalent to the speed at which the average propulsive force generated by a fin and the fluid resistance it encounters reach equilibrium. More simply, this velocity corresponds to the operational propulsion speed of the propulsion mechanism when it is set in self-propelled mode.

It should be noted that the average propulsive force of the fin was not measured immediately after changing the flow velocity in the water tunnel, but rather after waiting for the propulsive force to show periodic variations. Furthermore, accurately adjusting the flow speed within a water tank to achieve a mean propulsive force of zero generated by the propulsion mechanism is quite challenging. Hence, we determined the mean flow rate where the average propulsive force was around zero, with slight positive and negative propulsive forces occurring, and linearly approximated from there to find the flow rate at which the propulsive force became zero.

## 3. Results

### 3.1. Bending Resistance Characteristics of Fins

**Figure 7:**
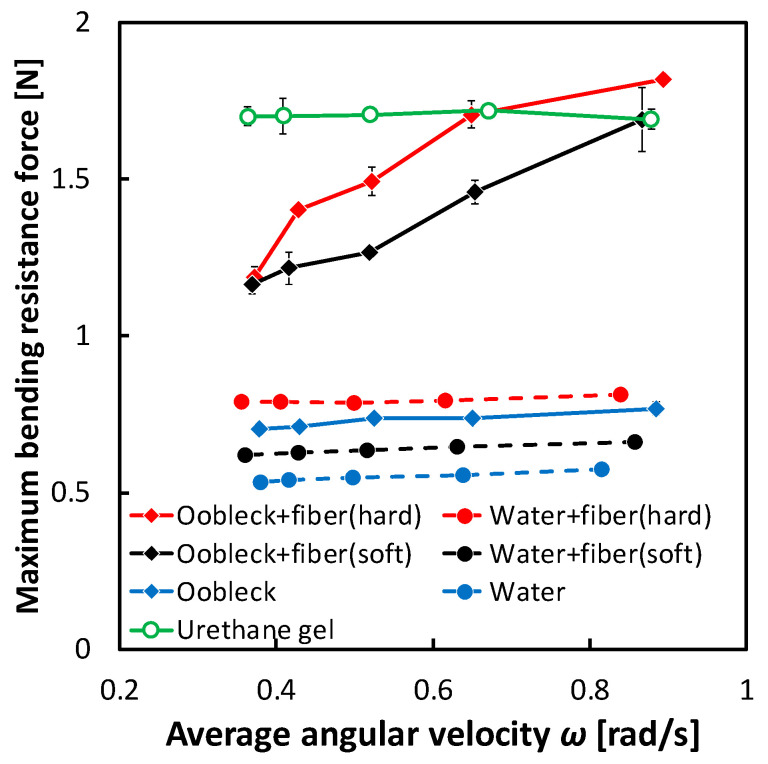
Relationship between average angular velocity ω and maximum bending resistance *F_max_*. Error bars indicate standard deviation (*n* = 5).

Figure 7 illustrates the dynamic interplay between the mean angular velocity ω and the maximum bending resistance *F_max_*. There exists a fluctuation within the scope of the oscillation angular velocity, ω, which is dependent upon the specific material constituting the fin. This irregularity can be traced back to the bending loads, which are inflicted upon the motor torque throughout the oscillation motion. One result of this variation is that maintaining a consistently average oscillation angular velocity becomes difficult, due to the fact that the maximum oscillation angle is always fixed at *θ*_max_ = 20°, regardless of any conceivable changes in the material of the fin.

From a more focused perspective, the fins constructed with a composition of oobleck and fibers demonstrated a noteworthy escalation in the *F_max_*, which corresponds with ω. This observation serves to show that the incremental increase in shear rate, particularly along the axis of the fiber direction in the shear thickening fluid, was instrumental in substantially enhancing the bending resistance of the fin. Taking this into consideration, the *F_max_* of fins that incorporated hard fibers consistently exceeded that of fins composed of soft fibers. On the other hand, when the fins were infused with water, their *F_max_* was lower. Furthermore, fins that were designed with an infusion of urethane gel presented an amplified *F_max_*. However, an intriguing point to note is that this amplified *F_max_* was predominantly invariant, with little to no deviation, despite the potential variations in ω.

### 3.2. Propulsion Characteristics in Still Water

#### 3.2.1. Thrust Force Characteristics

**Figure 8:**
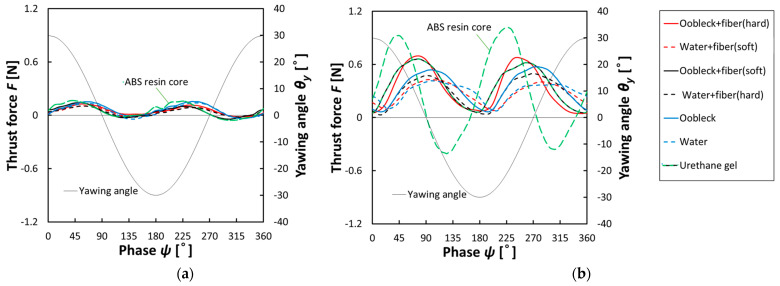
Relationship between thrust force and yawing phase (*θ_y-max_* = 30°, still water). (**a**) *T* = 3.5 s; (**b**) *T* = 1.5 s.

Figure 8 provides a visual representation of the variation in propulsion force observed over the duration of a single oscillation cycle in conditions of still water. This variation was seen in association with the oscillation angle. The data presented in this figure offer clear evidence that an increase in the length of the oscillation period, specifically at *T* = 3.5 s, led to a reduction in the propulsion force. Conversely, a decrease in the oscillation period, measured at *T* = 1.5 s, served to amplify the propulsion force. Moreover, it is particularly important to examine the role that the constituting material of the fin played during an oscillation period of *T* = 1.5 s. A fin equipped with a core made from ABS resin was capable of generating a significantly higher positive propulsion force but had a higher negative propulsion force compared to the other fin materials. By contrast, fins made from other materials, across the board, reliably produced a positive propulsion force. Of these, fins consisting of oobleck and hard fibers, along with the urethane gel fin, tended to generate notably higher positive propulsion forces.

**Figure 9:**
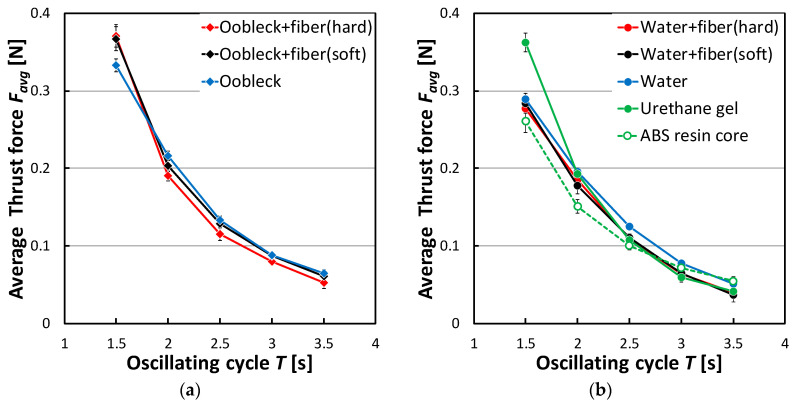
Relationship between average thrust force *F_avg_* and oscillating cycle *T* (*θ_y-max_* = 30°, still water). Error bars indicate standard deviation (*n* = 5). (**a**) Shear thickening fluid; (**b**) Newtonian fluid and other material.

Moving onto Figure 9, this displays the relationship that existed between the average thrust force *F_avg_* and the oscillation cycle *T*. Regardless of the specific conditions in question, *F_avg_* was observed to increase in value as *T* decreased. At *T* = 3.5 s, corresponding to the slowest rate of oscillation, the *F_avg_* associated with all fins under investigation was approximately equal. However, when the oscillation became quicker, at *T* = 1.5 s, the fins made from urethane gel and oobleck demonstrated a superior *F_avg_* production. Furthermore, when considering the fins containing oobleck, those that incorporated fibers displayed a larger *F_avg_* compared to those that did not, specifically at *T* = 1.5 s. The *F_avg_* associated with a fin containing oobleck and a softer fiber type matched or even exceeded that associated with the fins made from oobleck and hard fibers or urethane gel, across a broad spectrum of oscillation cycles. As a result of these findings, it is recommended that a fin containing oobleck and soft fibers is employed to sustain a high *F_avg_* over a wide range of oscillation cycles.

#### 3.2.2. Deformation Behavior

**Figure 10:**
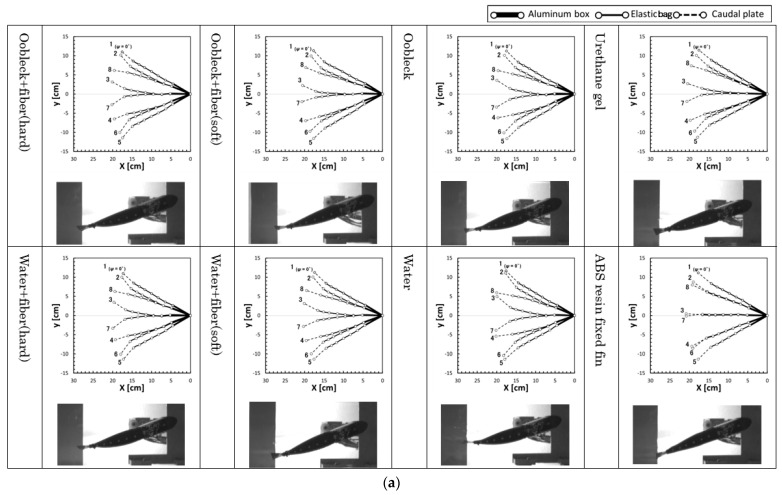

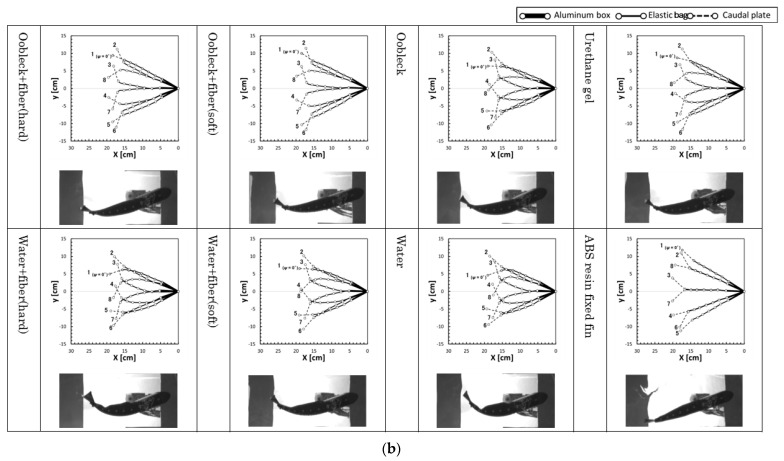
Yawing behavior of the fin (stick diagram of fin using markers shown in Figure 6) during an oscillation period (time step 1 to 8, 45° phase interval of oscillation) and picture of the fin at time step 4 (phase *ψ* = 135°) (*θ_y-max_* = 30°, still water). (**a**) *T* = 3.5 s; (**b**) *T* = 1.5 s.

Figure 10 illustrates the yawing behavior of the fin during an oscillation period and shows pictures of the fin. Two points in time, namely *T* = 3.5 s and *T* = 1.5 s, are specifically shown. The yawing behaviors of the fin shown in this figure are represented as stick diagrams, connecting the positions of the markers attached to the fin, as depicted in the right-hand side of Figure 6. The oscillation period is divided into eight equal parts, each representing a phase interval of 45 degrees. In addition, the pictures in the figure present detailed images of the fin during the fourth step of the time-sequence within the phase *ψ* = 135°. Additionally, to show the deformation behavior more clearly, we included a video of the movements of three types of different fin in Supplementary Video S1. This is a particular point where greater deformation of the fin is expected, and is typically significant. At the moment of *T* = 3.5 s, the deformation was notably pronounced in the fins that were filled with water-based compositions. The compositions in question here were water + fibers (hard), water + fibers (soft), and water. When compared to the other types of fin, these showed a more prominent deformation. In contrast, at the time point *T* = 1.5 s, it can be observed that the fins containing these water-based compositions—water + fibers (hard), water + fibers (soft), and water—demonstrated a distortion within the elastic bag section. This particular distortion was predominantly observed during the fourth time step and consequentially led the fin to acquire an irregular shape. Alternatively, fins that were filled with a mixture of oobleck and fibers, specifically oobleck + fibers (hard) and oobleck + fibers (soft), and those that are sealed with urethane gel, maintained their comprehensive structural integrity. They achieved this while exhibiting moderate deformation, as opposed to the significant distortion observed in the water-based fins. In the context of the ABS resin core, there was a noteworthy absence of deformation in the elastic bag section. The deformation only seemed to occur in the caudal plate, the tail-like structure. Based on the observations made, it is quite clear that it is of vital importance to maintain a moderate level of bending resistance in the elastic bag. This is because this plays a crucial role in generating an average propulsion force.

A comparison between fins filled with oobleck and fibers and those sealed with urethane gel shows that they exhibited similar or marginally greater deformations at *T* = 3.5 s, the slowest oscillation period. Furthermore, in the case of oobleck + fibers (hard) at *T* = 1.5 s, which was the fastest oscillation period, the deformation was either similar or marginally less. This suggests that fins constructed with a mixture of fibers and oobleck tended to become more rigid than those with elastic fins as the speed of motion increased. Consequently, they were able to sustain a high propulsion force even under varying speed conditions.

### 3.3. Propulsion Characteristics in Uniform Water Flow

**Figure 11:**
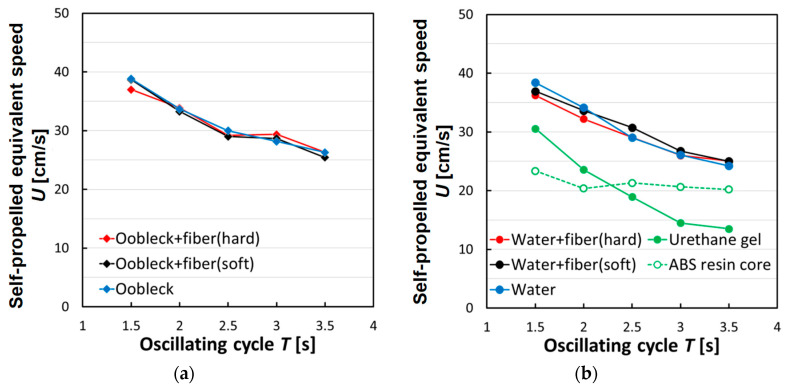
Relationship between self-propelled equivalent speed and bending cycle (*θ_y-max_* = 30°, uniform water flow). (**a**) Shear thickening fluid; (**b**) Newtonian fluid and other material.

Figure 11 illustrates the self-propelled equivalent speed for a single oscillation cycle within the specific period denoted as *T*, ranging between 1.5 and 3.5 s. An example of the results of the linear approximation used to calculate the average propulsive force for determining the self-propelled equivalent speed (under the conditions of oobleck + fibers (hard) *T* = 1.5 s, *θ_y-max_* = 30°) is shown in Supplementary Figure S1. In this representation, the fins that were filled with a non-Newtonian fluid, oobleck, did not present a consistently superior self-propelled equivalent speed in comparison with the other types of fin during all oscillation periods. In fact, their performance was nearly indistinguishable from that of the fins containing water. The fins filled with oobleck actually showed reduced values during the shorter oscillation period (*T* = 1.5 s), as opposed to the fins filled with water. This decrease in performance can likely be traced back to an excessive degree of rigidity inherent in the oobleck-filled fins, which may have hampered their effectiveness at such a rapid oscillation rate. Contrastingly, the fins sealed with a urethane gel, a material shown to provide relatively high propulsion in static water conditions, displayed very low self-propelled equivalent speeds when tested in a setting with a uniform water flow.

By examining these data points and experimental outcomes, we can make a few inferences. Fins sealed with a compliant urethane gel demonstrated a high propulsion capacity in static water conditions but fell short when it came to achieving high self-propelled equivalent speeds in uniform water flow. Conversely, fins filled with water, despite exhibiting a somewhat diminished propulsion in static water conditions, were seen to exhibit high self-propelled equivalent speeds when placed in uniform water flow. This highlights the potential of these fins to possess specific strengths and weaknesses, which became evident depending upon the operational environment they are subjected to. Interestingly, the fins containing oobleck seemed to defy this pattern. Despite the previously noted shortcomings, these fins demonstrated high propulsion in still water conditions and, intriguingly, they also managed to maintain high self-propelled equivalent speeds, even in the uniform water flow scenario. This indicates their intrinsic ability to uphold beneficial propulsive characteristics across a variety of environmental conditions.

## 4. Conclusions

We developed a viscoelastic fin integrated with a fiber composite shear thickening fluid (STF) using oobleck. Our findings demonstrated a correlation between the maximum bending resistance force and the rigidity of the fin, which was dependent on the applied oscillation period. In still water conditions, fins containing oobleck and soft fibers were able to achieve a higher average thrust than the other fins, regardless of the vibration period. In uniform flow conditions, although the self-propulsion equivalent speed of the fins containing oobleck and soft fibers was lower than that of fins containing water, it was higher than the other fins.

In the propulsion characteristic experiments conducted, the maximum yawing angle (*θ_y-max_*) was set to be constant at 30°. It would be necessary to vary this angle to further examine the propulsion characteristics. Furthermore, the periods of the oscillatory motion in the experiment were large, conducted within a range of slow oscillatory speeds; thus, it is necessary to also carry out experiments with faster oscillatory motion. It is important to perform these tests over a broader range, including the adjustment of the fiber volume ratio, the concentration of oobleck, and other related factors. Moreover, improving propulsion efficiency is important for realizing the propulsion mechanism, which also needs to be examined.

In the future, we will measure and evaluate the viscoelasticity of the fin component materials and the fin as a whole. The relationship between the shear rate and viscosity of the fiber and oobleck composite should also be determined and the influence of fiber elasticity should be discussed. In addition, a comprehensive analysis of the mechanical properties of the fins will be performed, taking into account the viscoelastic properties. We plan to evaluate the thrust force and the self-propelled equivalent speed in water with the developed fin. We used oobleck as a readily available shear thickening fluid. While it is a food ingredient and thus a biodegradable biomass material, it is not suitable for long-term use. Consequently, it is necessary to consider switching to other high-concentration granular suspensions [16] that possess shear thickening effects and that can be preserved over a long period. Additionally, it is also essential to explore other fibers, taking into account compatibility with suspensions and moving away from the current animal fibers.

## Figures and Tables

**Table 1:** Properties of fibers.

	Horse Hair (Hard)	Horse Hair (Soft)
Weight (tex: g/1000 m)	43.6	24.6
Average diameter in wet conditions (μm)	219	162
Resistance of incipient tension (N/tex)	2.52	1.82

**Table 2:** Types of fin used in the experiment (contents of the elastic bag).

Fibered/Unfibered	Contents of the Elastic Bag
Fibered *	60 wt% oobleck (shear thickening fluid) + hard fibers 60 wt% oobleck (shear thickening fluid) + soft fibers Water (Newtonian fluid) + hard fibers Water (Newtonian fluid) + soft fibers
Unfibered	60 wt% oobleck (shear thickening fluid) Water (Newtonian fluid) Urethane gel core (more elastic fin) ABS resin core (rigid fin)

* The fiber content in the elastic bag was 8.0 vol% for hard fibers and 6.0 vol% for soft fibers, respectively.

**Table 3:** Experimental conditions for propulsion characteristics.

Parameter	Range/Value
Oscillating cycle *T*	1.5–3.0 s
Yawing angle *θ_y_*	+/−30°, Amplitude *θ_y-max_* = 30°

## Data Availability

Not applicable.

## Supplementary Materials

The following supporting information can be downloaded at: https://www.mdpi.com/article/10.3390/biomimetics8050405/s1. Supplementary Video S1. Video of yawing behavior of the three fins: oobleck + fibers (hard), water, ABS resin core (*T* = 1.5 s, *θ_y-max_* = 30°). Supplementary Figure S1. An example of the relationship between the flow velocity in uniform flow and the average propulsive force (as the method of determining the self-propelled equivalent speed) (oobleck + fibers (hard), *T* = 1.5 s, *θ_y-max_* = 30°).
