# Supplementary material for: Bio-Inspired Aquatic Propulsion Mechanism Using Viscoelastic Fin Containing Fiber Composite Shear Thickening Fluid
Source: Biomimetics (Basel). 2023 Sep 1;8(5):405. doi: 10.3390/biomimetics8050405 (PMC10526487; doi:10.3390/biomimetics8050405)
Supplement: Supplementary file 1 [file biomimetics-08-00405-s001.zip › Supplementary S2.pdf]

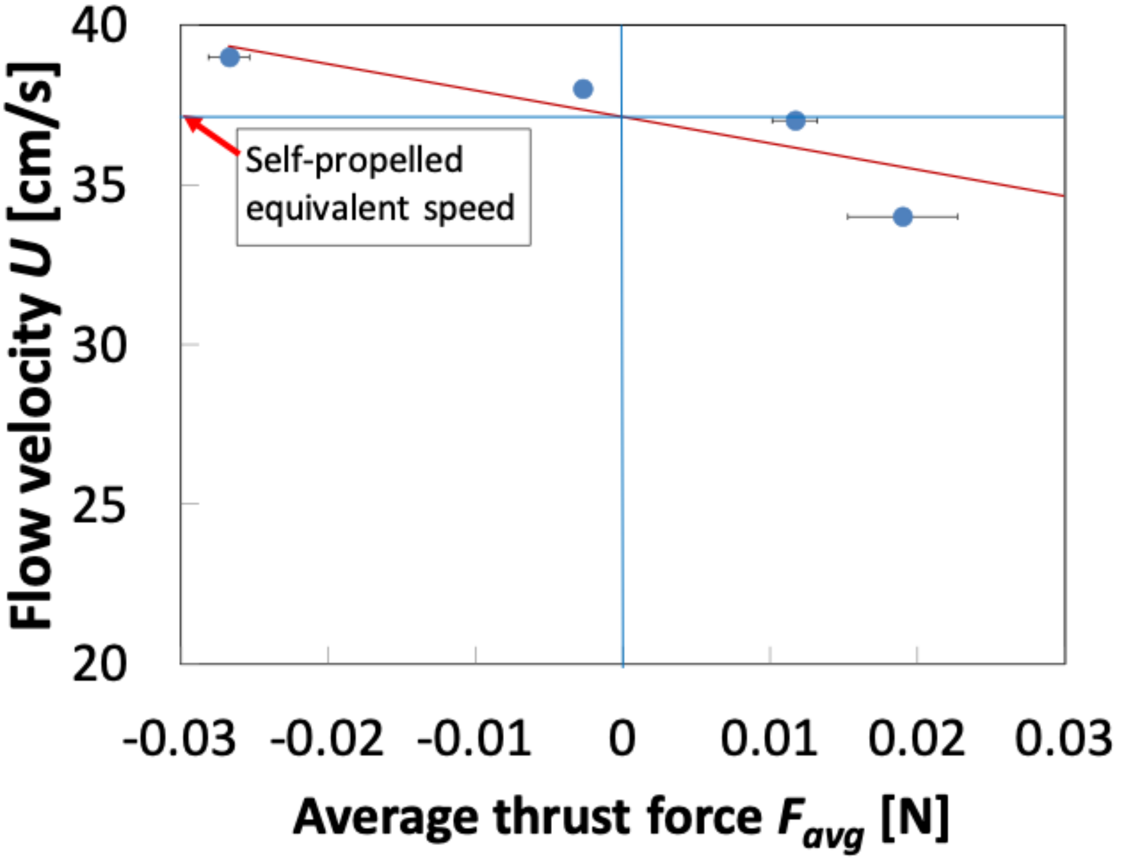

Appendix B: An example of the relationship between the flow velocity in uniform flow and the average propulsive force (as the method of determining the self-propelled equivalent speed) (oobleck + fibers (hard), T= 1.5s, $\theta_{y\text{-}max}$ = 30˚).
